# CMR-based aortic stiffness parameters in a symptomatic high risk obstructive sleep apnea cohort: results from the population-based Hamburg City Health Study

**DOI:** 10.1186/s12872-026-06450-z

**Published:** 2026-08-13

**Authors:** Katharina Alina Riedl, Konrad Reuter, Arne Böttcher, Markus Hüllebrand, Anja Hennemuth, Ersin Cavus, Rickmer Braren, Paulus Kirchhof, Stefan Blankenberg, Christian S. Betz, Gunnar Lund, Kai Muellerleile, Anna Sophie Hoffmann

**Affiliations:** 1 Department of Cardiology, University Heart and Vascular Center Hamburg, University Medical Center Hamburg, Hamburg, Germany; 2 German Center of Cardiovascular Research (DZHK), Partner Site Hamburg, Luebeck, Kiel, Hamburg, Germany; 3 Department of Cardiology, University Heart and Vascular Center Hamburg, Martinistraße 52, 20246 Hamburg, Germany; 4 Institute of Medical Technology and Intelligent Systems, Hamburg University of Technology, Hamburg, Germany; 5 Department of Otorhinolaryngology, University Medical Center Hamburg, Hamburg, Germany; 6 Deutsches Herzzentrum der Charité (DHZC), Berlin, Germany; 7 Charité-Universitätsmedizin Berlin, corporate member of Freie Universität Berlin and Humboldt-Universität zu Berlin, Berlin, Germany; 8 Fraunhofer Institute for Digital Medicine MEVIS, Berlin, Germany; 9 Department of Diagnostic and Interventional Radiology and Nuclear Medicine, University Medical Center Hamburg, Hamburg, Germany; 10 Chair for AI in Healthcare and Medicine, School of Medicine, TUM University Hospital, Technical University Munich, Munich, Germany

**Keywords:** Obstructive sleep apnea, Aortic stiffness, Pulse wave velocity, Aortic distensibility, Pulse pressure, Cardiovascular magnetic resonance imaging

## Abstract

**Background:**

Aortic stiffness (AS) parameters as pulse wave velocity (PWV), aortic distensibility of the ascending and descending aorta (AD AoAsc, AD AoDesc) and pulse pressure (PP) are marker for vascular ageing, whereas data concerning obstructive sleep apnea (OSA) in population-based cohorts are sparse. Thus, we aimed to identify possible associations of AS with OSA in the Hamburg City Health Study (HCHS) cardiovascular magnetic resonance (CMR) cohort.

**Methods:**

HCHS is a population-based cohort-study quantifying PWV and AD by 2D-phase-contrast-CMR-measuring-methods and PP by blood pressure. OSA was defined as self-reported OSA by HCHS-study-interviews and/or a combination of snoring, respiratory arrest and Epworth-Sleepiness-Scale (ESS) > 10. Logistic regression analyses with adjustments were performed.

**Results:**

In the cohort of 1,498 participants (median age 66 years, 39.0% female), 143 (9.5%) participants were identified as symptomatic high risk OSA cohort with significantly higher rates of males (*p* < 0.001), hypertension (*p* < 0.001), and coronary artery disease (*p* = 0.037). Median PWV and AD (*p* = 0.741, *p* = 0.145, *p* = 0.191) were not significantly different, but median PP was significantly higher in this OSA cohort (*p* = 0.003). In male OSA participants, median AD AoAsc and AD AoDesc were significantly lower and median PP significantly higher (*p* = 0.006, *p* = 0.015, *p* < 0.001). After adjustment for age, sex, cardiovascular risk factors (CVRF), diseases (CVD) and antihypertensive medication, OSA remained significantly associated with PP (*p* = 0.045), but not with PWV, AD AoAsc and AD AoDesc (*p* = 0.633, *p* = 0.503, *p* = 0.171).

**Conclusions:**

Higher PP was associated with OSA after adjustment for age, sex, CVRF, CVD and medication. Therefore, PP might be used to identify individuals at risk for OSA.

**Trial registration:**

The study has been registered at ClinicalTrial.gov (NCT03934957), retrospectively registered at 01/04/2019.

**Supplementary Information:**

The online version contains supplementary material available at 10.1186/s12872-026-06450-z.

## Background

Obstructive sleep apnea (OSA) describes a sleep disorder, characterized by recurrent upper airway obstruction during sleep, leading to respiratory interruptions and intermittent hypoxemia [1]. OSA remains largely undiagnosed in the general adult population [2]. Known risk factors to develop OSA are male sex, higher age and obesity [3]. OSA is a potentially life-threatening sleep disorder, due to discussed associations with cardiovascular diseases [4] with increased all-cause mortality [5], as well as with increased rates of psychiatric disorders [6]. The exact mechanism of the association between OSA and cardiovascular diseases is still not completely understood, while one hypothesis is the acceleration of vascular ageing. A marker for vascular ageing and atherosclerosis seems to be an increasing aortic stiffness (AS) [7], which can be quantified by pulse pressure (PP), using non-invasive blood pressure measurements, as well as by pulse wave velocity (PWV) and aortic distensibility (AD) using cardiovascular magnetic resonance (CMR) imaging. PP describes a non-invasive straightforward method using brachial blood pressure values and can be used as a surrogate marker for AS [8]. Moreover, PWV and AD describe stable parameter evaluating the individual aging of vessels [9, 10]. PWV increased with increasing blood pressure, age and male gender [11], which was confirmed in an analysis of aortic stiffness parameters in our Hamburg City Health Study (HCHS) CMR cohort [12]. Increasing aortic stiffness was described as a strong predictor of future cardiovascular events and all-cause mortality [13]. However, in the context of AS and OSA, the current literature reported contradicting results with [14, 15] and without associations between AS and OSA [16, 17]. In these studies, AS was quantified by different methods: by PP as a AS surrogate marker [15], by non-CMR-based carotid-femoral PWV (cfPWV) [14, 16] and by CMR-based PWV [17]. Data about aortic stiffness, especially including PP and CMR-based PWV and AD, as well as OSA data in high-volume population-based studies remains sparse [17]. Therefore, based on our data in the HCHS cohort [12] and the current knowledge about OSA and CVD, our objective was to examine possible associations between aortic stiffness parameters and OSA in this Hamburg City Health Study CMR cohort.

## Methods

### Study population and design

**Fig. 1:**
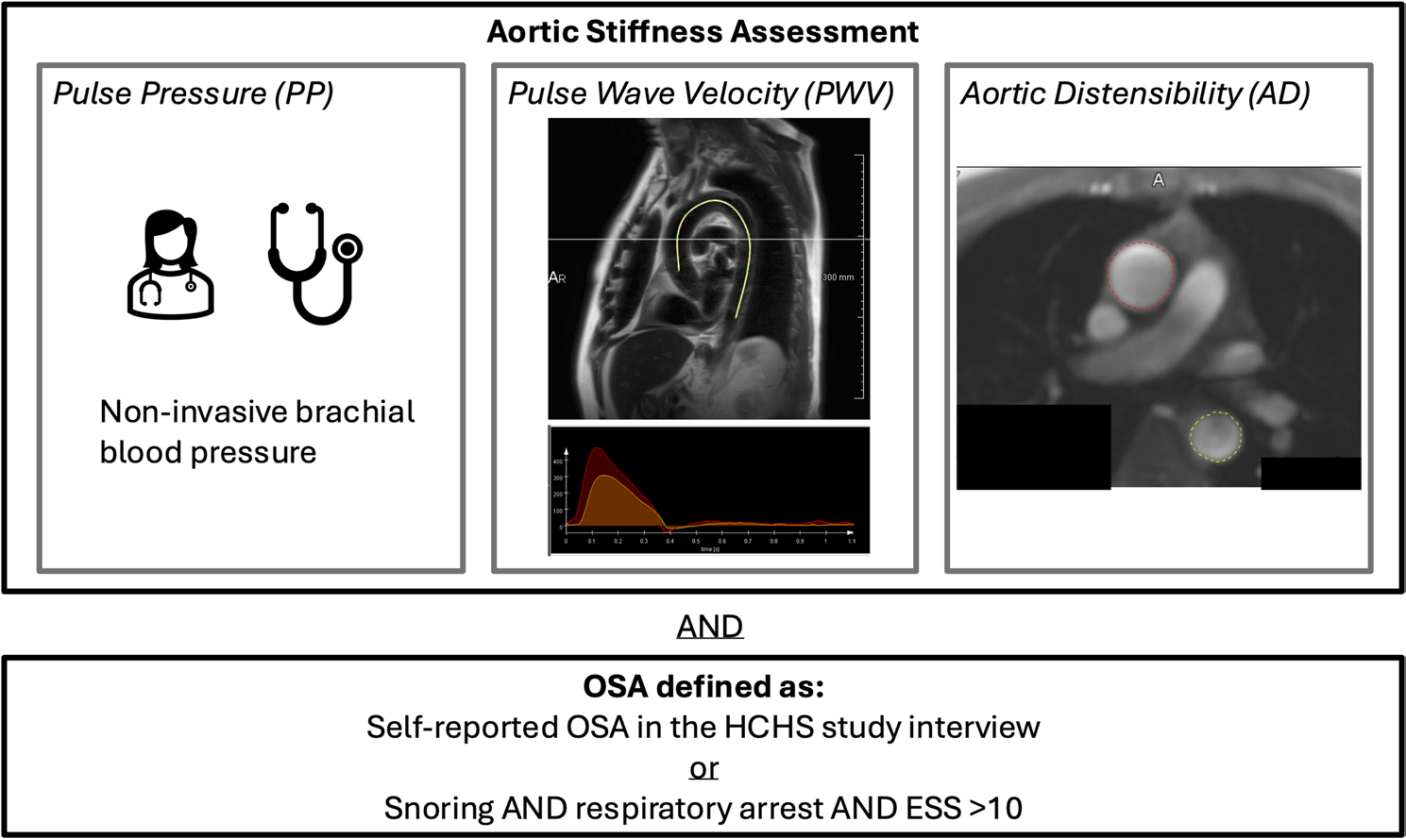
Methods. Aortic stiffness (AS) was quantified by pulse pressure (PP) using non-invasive brachial blood pressure values and by pulse wave velocity (PWV) and aortic distensibility (AD) quantified by cardiovascular magnetic resonance imaging. OSA was defined as self-reported OSA in the HCHS study interview or as a combination of snoring, respiratory arrest and ESS > 10. *HCHS = Hamburg City Health Study*,* CMR = cardiovascular magnetic resonance*,* OSA = obstructive sleep apnea*,* ESS = Epworth Sleepiness Scale*

The Hamburg City Health Study (HCHS) is a single-center, prospective, long-term, population-based, epidemiologic study with the aim to include over 30,000 citizens of the city of Hamburg, Germany, with an age between 45 and 74 years [18]. This analysis is based on the first available 10,000 participants of the HCHS, who were included between February 8th, 2016 and November 7th, 2018, of whom 1,498 participants underwent CMR-based AS quantification as previously described in [12, 19] and non-invasive blood pressure measurements for pulse pressure quantification, as well as exhibited available OSA data (Fig. 1, Supplemental Fig. 1). A symptomatic high risk OSA cohort was defined as self-reported OSA while HCHS study interview and/or as a combination of snoring with respiratory arrests and an Epworth Sleepiness Scale (ESS) > 10. The ESS conducted eight questions about the likelihood of falling asleep while sitting or reading, in front of the television, in the cinema or theatre, as a co-driver, while mid-day nap, sitting while talking, sitting while eating, in the car while a short stop [20] (Fig. 1).

### CMR protocols

All CMR scans were performed at a 3 Tesla magnetic resonance imaging (MRI) scanner (MAGNETOM™ Skyra, Siemens Healthineers, Erlangen, Germany) at the University Medical Center Hamburg-Eppendorf, Hamburg, Germany. Standardized CMR scan protocols were used as previously described [12, 19]. In particular, a parasagittal T2 HASTE (Half Fourier-acquired single shot turbo spin) sequence was performed, visualizing the whole aortic arch to obtain the exact distance ($\:\varDelta\:x$) between the two aortic measurement points in the ascending and descending aorta. Typical T2 HASTE MR parameters were: repetition time (TR) 3.8 ms, echo time (TE) 91 ms, flip angle (FA) 110°, voxel size 1.3 × 1.3 × 8 mm^3^, field of view (FoV) 400 mm^2^ with a parallel acquisition technique (PAT) factor of 2 [19]. Blood flow in the ascending and descending aorta was measured on a single slice using a 2D phase-contrast velocity-encoding (VENC) sequence at the level of the right pulmonary artery. Typical VENC-CMR parameters were: TR 4.3 ms, TE 2.3 ms, FA 20°, voxel size 1.2 × 1.2 × 8 mm^3^, FoV 300 mm^2^, PAT 2, 64 acquisitions per heart cycle [19].

### CMR-based PWV and AD analysis

CMR raw data were transferred to a dedicated, non-commercial analysis tool provided by the working group of Anja Hennemuth (Charité Berlin, Berlin, Germany) [21] to quantify PWV and AD values. All analyzes were performed and reviewed by a cardiologist with a level III certification for cardiac magnetic resonance imaging of the German Cardiac Society (KAR). PWV was defined as the distance between two measurement points divided by the transit time of the pulse wave between these two points [22]. The centerline method enables the measurement of the distance ($\:\varDelta\:x$) between the two measurement points in the ascending and descending aorta using the parasagittal T2 HASTE scan. Regions of interest were placed in the ascending and descending aorta to measure the transit time ($\:\varDelta\:t$) of the pulse wave between the ascending and descending aorta [22]. The PWV 50% algorithm uses the flow rate where it is half of the peak flow rate. This method was identified as a precise method for quantification PWV in population-based cohorts [12, 23–25]. AD was calculated dividing the relative change of the aortic diameter over the heart cycle by the pulse pressure (PP) according to Harloff et al. [12, 26]. Regions of interest (ROIs) were located in the ascending and descending aorta in all scans over the heart cycle measuring the relative change of the aortic diameter over time in [m]. The non-invasively brachial measured blood pressure was used to calculate the pulse pressure (PP), defined as the difference of the systolic and the diastolic blood pressure.

### Pulse pressure

Blood pressure was measured non-invasive brachial in all participants before the preformed CMR scan. Pulse pressure (PP) in [mmHg] is defined as the difference between systolic blood pressure and the diastolic blood pressure [27].

### Statistical analysis

Patients with incomplete responses to any of the questions defining OSA (self-reported OSA, snoring, respiratory arrests, ESS questions) were excluded from the study. In addition, only participants with high CMR image quality (exact CMR section planes and the absence of flow, breathing or motion artifacts impeding the image analyses) were included. Continuous variables are presented as median (25th, 75th percentile), and dichotomous variables as absolute and relative frequencies. Differences in continuous variables were evaluated using point-biserial correlation after Box-Cox transformation, whereas differences in dichotomous variables were assessed using chi-square test. For each questionnaire item, responses of “I don´t know” or “I prefer not to answer” were excluded from the respective analyses. To examine associations between aortic stiffness parameters (PWV, AD AoAsc, AD AoDesc, PP) and OSA, logistic regression models were constructed. Separate models were fitted for each stiffness parameter as the main independent variable, with OSA (yes/no) as the dependent variable. Models were adjusted for age, sex, obesity (BMI > 30 kg/m^2^), CVRF, CVD and antihypertensive medication. Stiffness parameters and age were included as continuous variables, while OSA, sex, obesity, CVRF, CVD and antihypertensive medication were treated as binary variables. Multicollinearity was assessed using variance inflation factors (VIFs). For each continuous predictor (PWV, AD AoAsc, AD AoDesc, PP) and for age within each corresponding model, we tested the linearity assumption by comparing a model including the linear term with a model including a restricted cubic spline term (4 degrees of freedom) via likelihood ratio test. A significance level of 0.05 was used for all analyses. All statistical analyses were performed using Python 3.12.7 with stasmodels 0.14.2 and SciPy 1.13.1. We used the STROBE reporting guideline to draft this manuscript [28].

## Results

### Study population

Our HCHS study cohort included 1,498 participants (median age 66 [58, 71] years, 39.0% female) (Supplemental Table 1, Supplemental Fig. 1). In this cohort, 143 participants fulfilled our criteria for a symptomatic high risk OSA cohort (Table 1, Supplemental Fig. 1). Participants in this OSA cohort were significantly less female (21.0% vs. 40.9%, *p* < 0.001) with higher median body mass index (BMI) (29.2 [26.1, 33.4] kg/m^2^ vs. 26.1 [23.8, 28.9] kg/m^2^, *p* < 0.001), higher rates of hypertension (62.2% vs. 40.0%, *p* < 0.001), and of coronary artery disease (CAD) (9.8% vs. 5.5%, *p* = 0.037) compared with the cohort without OSA (Table 1). Furthermore, in this cohort the rate of medication treatment with antihypertensive medication (58.6% vs. 40.1%, *p* < 0.001), angiotensin converting enzyme (ACE)- inhibitors and/or angiotensin II receptor blocker (ARB) (37.1% vs. 22.1%, *p* < 0.001), as well as with anti-platelet aggregatory medication (21.4% vs. 12.3%, *p* = 0.002) and aspirin (21.4% vs. 11.7%, *p* = 0.001) were significantly higher (Table 1). The rates of snoring (95.8% vs. 66.7%, *p* < 0.001) and respiratory arrests (92.3% vs. 7.6%, *p* < 0.001), as well as the number of participants with an ESS > 10 (37.8% vs. 10.6%, *p* < 0.001) were significantly higher in the OSA cohort compared with the cohort without OSA (Table 1).

**Table 1:** Study population according to OSA status

	CMR AS cohort *without* OSA (*n* = 1,355)	CMR AS cohort *with* OSA (*n* = 143)	*p*-value
Age [years]	66.0 [58.0, 71.0]	66.0 [60.0, 71.0]	0.546
Sex, females	554 (40.9)	30 (21.0)	< 0.001
Smoking (current and history)	884 (65.4)	99 (69.7)	0.300
BMI [kg/m^2^]	26.1 [23.8, 28.9]	29.2 [26.1, 33.4]	< 0.001
Systolic blood pressure before CMR [mmHg]	141.0 [128.0, 154.0]	140.0 [131.0, 152.0]	0.420
Diastolic blood pressure before CMR [mmHg]	82.5 [76.5, 90.0]	83.5 [76.0, 88.2]	0.678
Hypertension	536 (40.0)	89 (62.2)	< 0.001
Hyperlipidemia	344 (26.9)	44 (32.1)	0.191
Diabetes mellitus	120 (9.4)	16 (12.0)	0.335
Coronary artery disease	73 (5.5)	14 (9.8)	0.037
Myocardial infarction	43 (3.2)	8 (5.6)	0.206
Stroke	43 (3.2)	2 (1.4)	0.356
Heart failure	40 (3.7)	7 (5.9)	0.351
Creatinine [mg/dl]	0.87 [0.76, 0.99]	0.92 [0.80, 1.10]	0.002
Medication	1052 (80.6)	121 (86.4)	0.091
Antihypertensive medication	524 (40.1)	82 (58.6)	< 0.001
Diuretics	26 (2.0)	4 (2.9)	0.710
ACE inhibitor and/or ARB	289 (22.1)	52 (37.1)	< 0.001
Betablocker	219 (16.8)	27 (19.3)	0.451
Anti-platelet aggregatory therapy	160 (12.3)	30 (21.4)	0.002
Aspirin	153 (11.7)	30 (21.4)	0.001
Antidepressants	72 (5.5)	12 (8.6)	0.141
Snoring	904 (66.7)	137 (95.8)	< 0.001
*Snoring Frequency* Almost every day 3–5 times a week 1–2 times a week 1–2 times a month Less than once a month	250 (34.5) 169 (23.3) 191 (26.3) 68 (9.4) 47 (6.5)	101 (82.1) 11 (8.9) 8 (6.5) 3 (2.4) 0 (0.0)	< 0.001 < 0.001 < 0.001 0.017 0.007
Respiratory Arrest	103 (7.6)	132 (92.3)	< 0.001
*Respiratory Arrest Frequency* Almost every day 3–5 times a week 1–2 times a week 1–2 times a month Less than once a month	70 11 (15.7) 11 (15.7) 15 (21.4) 10 (14.3) 23 (32.9)	84 67 (79.8) 5 (6.0) 6 (7.1) 1 (1.2) 5 (6.0)	< 0.001 0.087 0.020 0.005 < 0.001
ESS > 10	143 (10.6)	54 (37.8)	< 0.001
*Aortic Stiffness Parameter*			
PWV [m/s]	7.45 [6.09, 9.64]	7.43 [6.15, 9.23]	0.741
AD AoAsc [1/(10^3*kPa)]	0.41 [0.26, 0.61]	0.37 [0.24, 0.57]	0.145
AD AoDesc [1/(10^3*kPa)]	0.44 [0.29, 0.65]	0.38 [0.27, 0.57]	0.191
PP [mmHg]	50.0 [42.0, 59.0]	53.0 [46.0, 63.0]	0.003

Continuous data are median and interquartile range. Categorical data are absolute numbers with percentages.

*CMR* cardiovascular magnetic resonance, *AS* aortic stiffness, *OSA* obstructive sleep apnea, *BMI* body mass index, *ACE* angiotensin converting enzyme, *ARB* angiotensin II receptor blocker, *PWV* pulse wave velocity, *AD* aortic distensibility, *AoAsc* ascending aorta, *AoDesc* descending aorta, *PP* pulse pressure, *ESS* Epworth Sleepiness Scale

### Aortic stiffness parameter

**Fig. 2:**
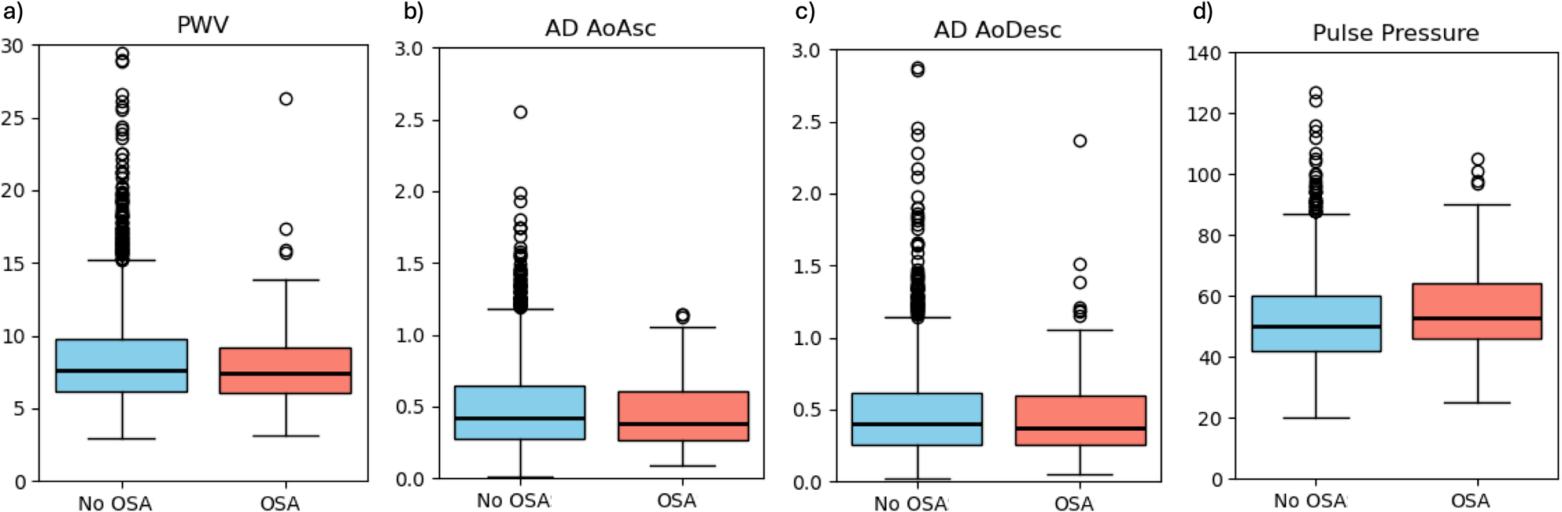
Boxplots PWV in [m/s] (*p* = 0.741) (a), AD AoAsc in [1/(10^3*kPa)] (*p* = 0.145) (b), AD AoDesc in [1/(10^3*kPa)] (*p* = 0.191) (c) and PP in [mmHg] (*p* = 0.003) according to OSA categories. *PWV = pulse wave velocity*,* AD = aortic distensibility*,* AoAsc = ascending aorta*,* AoDesc = descending aorta*,* PP = pulse pressure*,* OSA = obstructive sleep apnea*

PWV was 7.45 [6.10, 9.60] m/s, AD AoAsc 0.40 [0.26, 0.61] 1/(10^3*kPa), AD AoDesc 0.43 [0.28, 0.65] 1/(10^3*kPa), and PP 50.0 [42.0, 60.0] mmHg (Supplemental Table 1). PWV (7.43 [6.15, 9.23] m/s vs. 7.45 [6.09, 9.64] m/s, *p* = 0.741), AD AoAsc (0.37 [0.24, 0.57] 1/(10^3*kPa) vs. 0.41 [0.26, 0.61] 1/(10^3*kPa), *p* = 0.145), and AD AoDesc (0.38 [0.27, 0.57] 1/(10^3*kPa) vs. 0.44 [0.29, 0.65] 1/(10^3*kPa), *p* = 0.191) were not significantly different comparing participants with and without OSA (Table 1; Fig. 2). However, PP was significantly higher in the symptomatic high risk OSA cohort compared with the cohort without OSA (53.0 [46.0, 63.0] mmHg vs. 50.0 [42.0, 59.0] mmHg, *p* = 0.003) (Table 1; Fig. 2).

### Aortic stiffness parameter according to sex and age

In male participants of the symptomatic high risk OSA cohort, AD AoAsc (0.35 [0.24, 0.54] 1/(10^3*kPa) vs. 0.42 [0.27, 0.61] 1/(10^3*kPa), *p* = 0.006) and AD AoDesc (0.37 [0.25, 0.53] 1/(10^3*kPa) vs. 0.44 [0.30, 0.66] 1/(10^3*kPa), *p* = 0.015) were significantly lower and PP (53.0 [47.0, 64.0] mmHg vs. 49.0 [41.0, 58.0], *p* < 0.001) significantly higher compared with males without OSA (Table 2, Supplemental Fig. 2). PWV was not significantly different comparing male participants with and without OSA (*p* = 0.412) (Table 2, Supplemental Fig. 2). Comparing female participants with and without symptoms and high risk for OSA, no significant differences could be found (PWV: *p* = 0.123, AD AoAsc: *p* = 0.277, AD AoDesc: *p* = 0.583, PP: *p* = 0.706) (Table 2, Supplemental Fig. 2). In this OSA cohort of participants with an age ≥ 65 years, PP (59.0 [48.0, 64.0] mmHg vs. 53.0 [45.0, 63.0] mmHg, *p* = 0.041) was significantly higher compared with participants without OSA (Supplemental Table 2). Formal sex interaction tests showed no significant effect modification for AD AoAsc (*p* = 0.055) or AD AoDesc (*p* = 0.081). However, the PP-by-sex interaction was nominally significant (*p* = 0.048), indicating potential sex-specific effect modification. In the remaining age categories, no significant differences of PWV, AD AoAsc, AD AoDesc, and PP were found comparing participants with and without OSA (Supplemental Table 2). An age interaction test for PP showed no effect modification (≥ 65 years old vs. <65 years old; *p* = 0.435).

**Table 2:** Study population according to OSA and sex categories

	CMR AS cohort *without* OSA MALE (*n* = 801)	CMR AS cohort *with* OSA MALE (*n* = 113)	*p*-value	CMR AS cohort *without* OSA FEMALE (*n* = 554)	CMR AS cohort *with* OSA FEMALE (*n* = 30)	*p*-value
Age [years]	66.0[59.0, 71.0]	66.0[60.0, 71.0]	0.450	65.0[57.0, 71.0]	64.0[58.0, 68.0]	0.572
Smoking (current and history)	559 (69.8)	82 (73.2)	0.458	325 (59.0)	17 (56.7)	0.802
BMI [kg/m^2^]	26.3 [24.4, 28.8]	29.2 [26.1, 32.7]	< 0.001	25.7 [22.9, 29.3]	29.3 [26.4, 35.7]	< 0.001
Systolic blood pressure before CMR [mmHg]	142.0 [130.0, 155.0]	141.0 [132.0, 153.0]	0.575	138.0 [126.0, 152.0]	136.0 [128.0, 144.0]	0.508
Diastolic blood pressure before CMR [mmHg]	83.5 [76.5, 91.0]	83.0 [76.6, 88.5]	0.405	81.5 [75.5, 88.0]	83.5 [74.0, 85.0]	0.685
Hypertension	319 (40.3)	71 (62.8)	< 0.001	217 (39.5)	18 (60.0)	0.026
Hyperlipidemia	228 (30.3)	37 (34.3)	0.407	116 (22.0)	7 (24.1)	0.965
Diabetes mellitus	74 (9.8)	13 (12.4)	0.417	46 (8.9)	3 (10.7)	1.000
Coronary artery disease	52 (6.6)	13 (11.5)	0.058	21 (3.9)	1 (3.3)	1.000
Myocardial infarction	30 (3.8)	8 (7.1)	0.160	13 (2.4)	0 (0.0)	0.827
Stroke	28 (3.5)	2 (1.8)	0.490	15 (2.7)	0 (0.0)	0.763
Heart failure	26 (4.1)	5 (5.4)	0.764	14 (3.2)	2 (8.0)	0.465
Creatinine [mg/dl]	0.95 [0.86, 1.10]	0.97 [0.85, 1.10]	0.806	0.76 [0.68, 0.84]	0.79 [0.70, 0.88]	0.220
Medication	582 (76.0)	94 (84.7)	0.041	470 (87.0)	27 (93.1)	0.502
Antihypertensive medication	311 (40.6)	64 (57.7)	< 0.001	213 (39.4)	18 (62.1)	0.016
Diuretics	10 (1.3)	3 (2.7)	0.473	16 (3.0)	1 (3.5)	1.000
ACE inhibitor and/or ARB	186 (24.3)	42 (37.8)	0.002	103 (19.1)	10 (34.5)	0.074
Betablocker	117 (15.3)	21 (18.9)	0.324	102 (18.9)	6 (20.7)	1.000
Anti-platelet aggregatory therapy	113 (14.8)	28 (25.2)	0.005	47 (8.7)	2 (6.9)	1.000
Aspirin	111 (14.5)	28 (25.2)	0.004	42 (7.8)	2 (6.9)	1.000
Antidepressants	27 (3.5)	8 (7.2)	0.111	45 (8.3)	4 (13.8)	0.496
Snoring	589 (73.5)	108 (95.6)	< 0.001	315 (56.9)	29 (96.7)	< 0.001
*Snoring Frequency* Almost every day 3–5 times a week 1–2 times a week 1–2 times a month Less than once a month	173 (34.3) 133 (26.4) 135 (26.8) 40 (7.9) 23 (4.6)	79 (79.8) 10 (10.1) 8 (8.1) 2 (2.0 0 (0.0)	< 0.001 < 0.001 < 0.001 0.058 0.060	77 (34.8) 36 (16.3) 56 (25.3) 28 (12.7) 24 (10.9)	22 (91.7) 1 (4.2) 0 (0.0) 1 (4.2) 0 (0.0)	< 0.001 0.202 0.011 0.372 0.181
Respiratory Arrest	83 (10.4)	104 (92.0)	< 0.001	20 (3.6)	28 (93.3)	< 0.001
*Respiratory Arrest Frequency* Almost every day 3–5 times a week 1–2 times a week 1–2 times a month Less than once a month	8 (13.8) 9 (15.5) 13 (22.4) 7 (12.1) 21 (36.2)	51 (77.3%) 4 (6.06%) 6 (9.09%) 1 (1.52%) 4 (6.06%)	< 0.001 0.155 0.071 0.043 < 0.001	3 (25.0) 2 (16.7) 2 (16.7) 3 (25.0) 2 (16.7)	16 (88.9) 1 (5.6) 0 (0.0) 0 (0.0) 1 (5.6)	0.002 0.709 0.296 0.106 0.709
ESS > 10	82 (10.2)	43 (38.1)	< 0.001	61 (11.0)	11 (36.7)	< 0.001
*Aortic stiffness parameter*						
PWV [m/s]	7.35 [6.17, 9.44]	7.91 [6.48, 9.28]	0.412	7.55 [5.98, 9.79]	6.18 [5.63, 8.21]	0.123
AD AoAsc [1/(10^3*kPa)]	0.42 [0.27, 0.61]	0.35 [0.24, 0.54]	0.006	0.39 [0.25, 0.64]	0.46 [0.31, 0.72]	0.277
AD AoDesc [1/(10^3*kPa)]	0.44 [0.30, 0.66]	0.37 [0.25, 0.53]	0.015	0.44 [0.27, 0.65]	0.46 [0.28, 0.65]	0.583
PP [mmHg]	49.0 [41.0, 58.0]	53.0 [47.0, 64.0]	< 0.001	50.0 [42.0, 62.0]	50.0 [43.0, 59.5]	0.706

Continuous data are median and interquartile range. Categorical data are absolute numbers with percentages.

*CMR* cardiovascular magnetic resonance, *AS* aortic stiffness, *OSA* obstructive sleep apnea, *BMI* body mass index, *ACE* angiotensin converting enzyme, *ARB* angiotensin II receptor blocker, *PWV* pulse wave velocity, *AD* aortic distensibility, *AoAsc* ascending aorta, *AoDesc* descending aorta, *PP* pulse pressure, *ESS* Epworth Sleepiness Scale

### Aortic stiffness and OSA

**Fig. 3:**
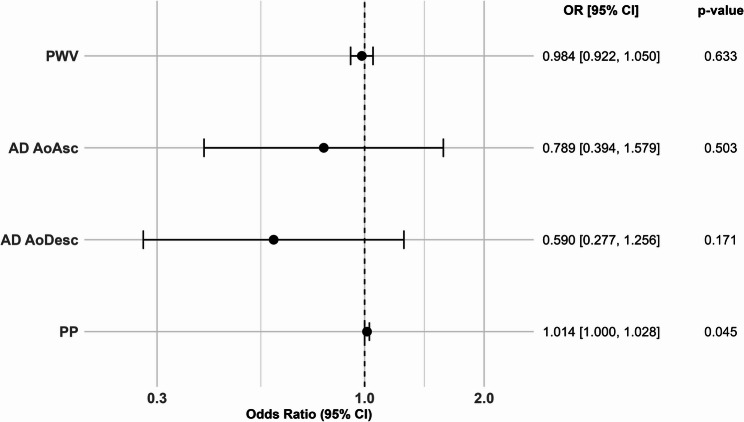
Forest plot associations between PWV, AD AoAsc, AD AoDesc and PP and OSA, after adjustment for age, sex, CVRF, CVD and antihypertensive medication. *PWV = pulse wave velocity*,* AD = aortic distensibility*,* AoAsc = ascending aorta*,* AoDesc = descending aorta*,* PP = pulse pressure*,* OSA = obstructive sleep apnea*

Regression analyses without any adjustments were reported in Supplemental Table 3. Data after adjustment for age and sex were summarized in Supplemental Table 4 and after adjustment for age, sex, obesity (BMI > 30 kg/m^2^) and hypertension in Supplemental Table 5. After adjustment for age, sex, CVRF, CVD and with antihypertensive medication, OSA remained significantly associated with PP (OR 1.014 [1.000, 1.028], *p* = 0.045), but not with PWV (*p* = 0.633), AD AoAsc (*p* = 0.503) and AD AoDesc (*p* = 0.171) (Table 3; Fig. 3). The effect of the adjustment was predominantly driven by sex and obesity (*p* < 0.001) (Table 3). Multicollinearity was assessed using variance inflation factors (VIFs). Slightly elevated VIFs were observed for hypertension and antihypertensive medication, but all VIFs remained well below the commonly used threshold of 5 (Supplemental Table 6). For each continuous predictor (PWV, AD AoAsc, AD AoDesc, and PP), as well as age within the corresponding model, the linearity assumption was assessed by comparing models including linear terms with models including restricted cubic spline terms with 4 degrees of freedom using likelihood ratio tests. None of the likelihood ratio tests indicated a statistically significant deviation from linearity (all *p* > 0.05) (Supplemental Table 7).

**Table 3:** Regression analysis of OSA with PWV, AD and PP after adjustment for age, sex, cardiovascular risk factors and diseases and antihypertensive medication

	PWV model		AD AoAsc model		AD AoDesc model		PP model	
	OR [95% CI]	*p*-value	OR [95% CI]	*p*-value	OR [95% CI]	*p*-value	OR [95% CI]	*p*-value
PWV/AD/PP variable	0.984 [0.922, 1.050]	0.633	0.789 [0.394, 1.579]	0.503	0.590 [0.277, 1.256]	0.171	1.014 [1.000, 1.028]	0.045
Age	0.997 [0.971, 1.024]	0.818	0.990 [0.965, 1.017]	0.471	0.988 [0.963, 1.013]	0.342	0.992 [0.967, 1.018]	0.563
Sex	0.377 [0.242, 0.588]	< 0.001	0.378 [0.242, 0.589]	< 0.001	0.379 [0.243, 0.592]	< 0.001	0.364 [0.228, 0.580]	< 0.001
Obesity (BMI > 30 kg/m^2^)	3.167 [2.129, 4.711]	< 0.001	3.153 [2.120, 4.688]	< 0.001	3.079 [2.068, 4.585]	< 0.001	2.923 [1.929, 4.430]	< 0.001
Hypertension	2.166 [1.180, 3.977]	0.013	2.153 [1.172, 3.952]	0.013	2.108 [1.146, 3.879]	0.016	1.660 [0.877, 3.143]	0.119
Hyperlipidemia	0.917 [0.589, 1.427]	0.700	0.918 [0.590, 1.428]	0.703	0.917 [0.589, 1.426]	0.699	0.988 [0.629, 1.553]	0.960
Smoking	1.001 [0.982, 1.020]	0.909	1.001 [0.978, 1.025]	0.924	1.001 [0.976, 1.027]	0.930	1.001 [0.971, 1.032]	0.940
Diabetes mellitus	0.916 [0.494, 1.696]	0.779	0.922 [0.499, 1.705]	0.796	0.914 [0.493, 1.693]	0.775	0.862 [0.453, 1.640]	0.651
CAD	1.348 [0.639, 2.845]	0.433	1.366 [0.649, 2.876]	0.411	1.396 [0.662, 2.942]	0.380	1.425 [0.675, 3.007]	0.353
Myocardial infarction	1.293 [0.508, 3.292]	0.590	1.285 [0.505, 3.267]	0.599	1.311 [0.516, 3.333]	0.570	1.296 [0.507, 3.314]	0.588
Antihypertensive medication	0.918 [0.498, 1.692]	0.784	0.926 [0.503, 1.707]	0.806	0.954 [0.516, 1.762]	0.880	1.065 [0.560, 2.023]	0.848

*OSA* obstructive sleep apnea, *PWV* pulse wave velocity, *AD* aortic distensibility, *AoAsc* ascending aorta, *AoDesc* descending aorta, *OR* odds ratio, *PP* pulse pressure, *BMI* body mass index, *CAD* coronary artery disease

## Discussion

Our large HCHS CMR study cohort (*n* = 1,498) enabled the assessment of PP, CMR-based PWV and AD, as well as data about OSA, defined as self-reported OSA in the HCHS study interview or snoring with respiratory arrest and an ESS > 10, in the same study population. The three main findings of this analysis were:

1. Pulse pressure was significantly higher in the symptomatic high risk OSA cohort compared with participants without symptoms and a high risk for OSA.
2. AD AoAsc and AD AoDesc was significantly lower and PP significantly higher in male participants in the symptomatic high risk OSA cohort compared with male participants without symptoms and a high risk for OSA.
3. After adjustment for age, sex, obesity, CVRF, CVD and antihypertensive medication, OSA was significantly associated only with higher PP values.

### OSA and cardiovascular risk factors and diseases

As excepted, OSA was reported in the HCHS CMR cohort in more male participants with higher BMI and higher rates of cardiovascular risk factors and diseases, like hypertension and CAD (Table 1). This is in accordance with recent studies, in which OSA was predominantly documented in male [14, 29] and obese patients [30] with chronic disorders like hypertension [31]. Furthermore, in a Scandinavian study, OSA was also found in more male patients with a higher BMI and significant higher numbers of hypertension [15]. Korcarz et al. reported a significant higher number of antihypertensive medications in their sleep disordered breathing (SDB) group with 44.6%, which was also documented in our HCHS OSA cohort with a treatment with antihypertensive medication in 58.6% of the OSA participants and with ACE inhibitors and/or ARB in 37.1% [32]. These data confirmed the dominance of hypertension and CVD with a high number of corresponding antihypertensive and cardiovascular-treatment medication in HCHS OSA participants (Table 1). Especially secondary hypertension can be caused by OSA due to the hypoxemia and oxidative stress reactions, which can also lead to increased aortic stiffness [15, 33]. Controversially to our data, studies reported OSA more common in older patients, which might be attributed to changes in the upper airways and the respiratory muscles [14, 34]. In our HCHS CMR cohort, the median age did not differ significantly comparing the cohort with and without OSA. This could be explained by the population-based setting of the HCHS including participants between 45 and 74 years with a median age of 66 years in the HCHS CMR cohort (Table 1). The prevalence of OSA in our HCHS CMR cohort (9.55%, 143/1,498) is within the range of OSA prevalences found in a meta-analysis of population-based studies with 6–17% [35, 36]. Furthermore, the HCHS described a population-based cohort study without polysomnography data for OSA diagnosis. Overall, the analyzed HCHS CMR cohort depicted a representative cohort for OSA, expanding the data of the current literature [37].

### Aortic stiffness in OSA patients

In the HCHS, pulse pressure was significantly higher in participants in the symptomatic high risk OSA cohort (Table 1, Fig. 2), as well as AD AoAsc and AD AoDesc significantly lower and PP significantly higher in male participants in the symptomatic high risk OSA cohort (Table 2, Supplemental Fig. 2). A large-scale Scandinavian study including over 6,000 participants with 34.5% OSA patients demonstrated significantly higher pulse pressure values in the OSA cohort [15]. These data are in accordance with our data with significant higher PP values in the symptomatic high risk OSA cohort and also in the male OSA sub-cohort. However, the male-to-female ratio in our cohort was not balanced with a small number of female participants in the OSA cohort. The PP-by-sex interaction was nominally significant (*p* = 0.048), suggesting potential sex-specific effect modification. Given the limited number of females with OSA, this finding should be interpreted cautiously and warrants confirmation in larger cohorts. Moreover, Jenner et al. analyzed carotid-femoral PWV (cfPWV) in 95 patients (54.7% OSA patients) with significantly higher cfPWV values in OSA patients, also after stratification by sex [14]. Additionally, in the Maastricht Study, cfPWV was measured in low risk OSA and high risk OSA participants (*n* = 1,352 vs. *n* = 423) with higher cfPWV values in the high-risk group. Whereas the association with cfPWV was no longer significant after adjustment for mean blood pressure [38]. These data are contradicting to our PWV data without a significant difference, whereas the rate of OSA participants was lower in our cohort and PWV was quantified CMR-based. In contrast, the MESA study (*n* = 708) also performed CMR scans to quantify PWV without significant differences of PWV after adjustment for potential confounders [17], which is in line with our results. These data were also confirmed by Korcarz CE et al. investigating objects with and without sleep disordered breathing (SDB) (*n* = 83 and 70). They found no significant differences of the PWV values quantified by arterial tonometry [32]. Several studies could demonstrate that the cfPWV method in contrast to the CMR-based method does not include the exact distance between the carotid and femoral measurement points without taking into consideration the ascending aorta and the aortic arch [39]. These discrepance of the methods can also explain the contradicting results. However, the HCHS does not provide data about the risk of OSA, the severity and the treatment of OSA. Therefore, further studies investigating risk categories for OSA, especially in the context with CMR-based AS measurements, are needed.

### Associations between OSA and increased aortic stiffness

In our analysis, OSA was significantly associated only with higher PP after adjustment for age, sex, CVRF, CVD and antihypertensive medication (Table 3, Fig. 3). The current literature provided contradicting results regarding associations between OSA and arterial stiffness parameters: Higher PWV [14] and higher PP values [15] were found in OSA patients, whereas contradicting studies demonstrated no verifiable associations [17, 32, 38]. Most of the studies investigating associations between OSA and arterial stiffness used PP as a surrogate marker for increased arterial stiffness [15]. One strength of the HCHS comprised the measurement of different AS parameters in the same cohort: the direct, exact non-invasive CMR-based PWV and AD assessment [40] as well as the quantification of PP as a non-invasive surrogate marker for AS [41]. To the best of our knowledge, data comparing CMR-based AD values in a OSA cohort are missing so far and firstly analyzed in our cohort. Our data suggested that PP might be a possible biomarker for OSA risk stratification. PP serves as a simple marker reflecting “global” aortic stiffness, cardiac output and hyper-sympathetic nervous system activation [42]. In contrast, PWV and AD quantify regional, structural aortic stiffness. While factors like male sex, obesity and hypertension might drive wall remodeling, PP is highly responsive to the dynamic stress and hyperdynamic episodes directly triggered by OSA [15]. Therefore, PP seems to represent a simple surrogate marker to depict individuals at risk for OSA, which could be used as a gatekeeper for time-consuming OSA screening. However, this topic requires a dedicated clinical study. Furthermore, our findings expand current literature on potential interactions between aortic stiffness and OSA, but do not allow proof of causality, which is beyond the scope of a cross-sectional, population-based study.

### Strengths and limitations

One limitation compared to other studies is that OSA was defined as self-reporting OSA in the HCHS study interview or a combination of snoring, respiratory arrests and an ESS > 10. This OSA assessment was based on subjective evaluation, which is validated as a screening tool. Therefore, our cohort represents participants with a high risk for the presence of OSA. The HCHS is a population-based cohort study without the aim of polysomnography-based OSA testing in all participants which is beyond the scope of a cross-sectional population-based study. Additionally, the assessment of excessive daytime sleepiness using the Epworth Sleepiness Scale can potentially encompass a phenotype present in multiple sleep disorders. Nevertheless, this provides a main strength of our analyses, and our data can strengthen the role of PP in OSA screening and risk stratification. Further data about the deeper exploration of pathophysiology and inter play between OSA-induced stress and chronic vascular remodeling are not available in the HCHS and beyond the scope of this population-based cohort study setting and have to be fully addressed in further clinical and/or experimental studies. Furthermore, the male-to-female-ratio was not completely balanced in the HCHS CMR cohort, which is in accordance with other CMR-based AS studies [8, 43] and with the known prevalence of OSA in females in the general population [44]. A potential technical limitation is the use of a 2D flow CMR method in this study in contrast to available 4D flow CMR methods. However, the majority of studies assessing aortic stiffness and OSA did not perform CMR, whereas CMR and especially a 2D flow CMR approach is better suited for clinical CMR protocols due to fast data acquisition and analyses, especially in large population-based studies and can assess PWV and AD direct and exact [45]. Furthermore, we have to point out limitations of multiple testing, whereas available correction methods of multiple testing also exhibit additional problems, as routine adjustment for multiple comparisons may increase the risk of overlooking true associations (type II error) [46]. Of note, PWV, AD, and PP are dynamic parameters, which can be influenced by changes in medication and/or physical activity [47], but an analysis of these potential confounders is beyond the scope of this cross-sectional, population-based study. Several studies focused on the change of AS parameters in the context of OSA treatment with reduced aortic stiffness, especially PWV, in OSA patients [48]. In our study, currently no data about treatment or follow-up data are available.

## Conclusions

After adjustment for age, sex, obesity, CVRF, CVD and antihypertensive medication, OSA was significantly associated only with higher PP. Therefore, PP might be used to identify individuals at risk for OSA considering that PP describes a non-specific marker. Further detailed studies are needed to address this finding. 

## Supplementary Information

Supplementary Material 1.

Supplementary Material 2.

## Data Availability

The datasets used and/or analyzed during the current study are available from the corresponding author on reasonable request.

## Abbreviations

AD: Aortic distensibility
AoAsc: Ascending aorta
AoDesc: Descending aorta
AS: Aortic stiffness
CfPWV: Carotid femoral pulse wave velocity
CMR: Cardiovascular magnetic resonance
CVD: Cardiovascular disease
CVRF: Cardiovascular risk factor
HCHS: Hamburg City Health Study
OSA: Obstructive sleep apnea
PP: Pulse pressure
PWV: Pulse wave velocity

## References

[1] Sánchez-de-la-Torre M, Campos-Rodriguez F, Barbé F. Obstructive sleep apnoea and cardiovascular disease. Lancet Respir Med. 2013;1(1):61–72. 24321805 10.1016/S2213-2600(12)70051-6

[2] Young T, Evans L, Finn L, Palta M. Estimation of the clinically diagnosed proportion of sleep apnea syndrome in middle-aged men and women. Sleep. 1997;20(9):705–6. 9406321 10.1093/sleep/20.9.705

[3] Young T, Shahar E, Nieto FJ, Redline S, Newman AB, Gottlieb DJ, et al. Predictors of sleep-disordered breathing in community-dwelling adults: the Sleep Heart Health Study. Arch Intern Med. 2002;162(8):893–900. 11966340 10.1001/archinte.162.8.893

[4] Somers VK, White DP, Amin R, Abraham WT, Costa F, Culebras A, et al. Sleep Apnea Cardiovasc Disease Circulation. 2008;118(10):1080–111. 10.1161/CIRCULATIONAHA.107.189375 18725495

[5] Redline S, Azarbarzin A, Peker Y. Obstructive sleep apnoea heterogeneity and cardiovascular disease. Nat Rev Cardiol. 2023;20(8):560–73. 36899115 10.1038/s41569-023-00846-6

[6] Dai Y, Li X, Zhang X, Wang S, Sang J, Tian X, et al. Prevalence and Predisposing Factors for Depressive Status in Chinese Patients with Obstructive Sleep Apnoea: A Large-Sample Survey. PLoS ONE. 2016;11(3):e0149939. 26934192 10.1371/journal.pone.0149939 PMC4774961

[7] Yim-Yeh S, Rahangdale S, Nguyen AT, Jordan AS, Novack V, Veves A, et al. Obstructive sleep apnea and aging effects on macrovascular and microcirculatory function. Sleep. 2010;33(9):1177–83. 20857864 10.1093/sleep/33.9.1177 PMC2938858

[8] Said MA, Eppinga RN, Lipsic E, Verweij N, van der Harst P. Relationship of Arterial Stiffness Index and Pulse Pressure With Cardiovascular Disease and Mortality. J Am Heart Association. 2018;7(2). 10.1161/JAHA.117.007621 PMC5850166 29358193

[9] Smulyan H, Mookherjee S, Safar ME. The two faces of hypertension: role of aortic stiffness. J Am Soc Hypertens. 2016;10(2):175–83. 26725014 10.1016/j.jash.2015.11.012

[10] Cecelja M, Ruijsink B, Puyol-Antón E, Li Y, Godwin H, King AP, et al. Aortic Distensibility Measured by Automated Analysis of Magnetic Resonance Imaging Predicts Adverse Cardiovascular Events in UK Biobank. J Am Heart Association. 2022;11(23):e026361. 10.1161/JAHA.122.026361 PMC9851433 36444831

[11] Harloff A, Mirzaee H, Lodemann T, Hagenlocher P, Wehrum T, Stuplich J, et al. Determination of aortic stiffness using 4D flow cardiovascular magnetic resonance - a population-based study. J Cardiovasc Magn resonance: official J Soc Cardiovasc Magn Reson. 2018;20(1):43. 10.1186/s12968-018-0461-z PMC6011486 29925388

[12] Riedl KA, Di Carluccio E, Huellebrand M, Hennemuth A, Frye M, Kaufmann P et al. Associations between cardiovascular risk factors and diseases with aortic pulse wave velocity and aortic distensibility: magnetic resonance imaging in the Hamburg city health study. Clin Res Cardiol. 2026. 10.1007/s00392-026-02866-x PMC13346220 41661257

[13] Vlachopoulos C, Aznaouridis K, O’Rourke MF, Safar ME, Baou K, Stefanadis C. Prediction of cardiovascular events and all-cause mortality with central haemodynamics: a systematic review and meta-analysis. Eur Heart J. 2010;31(15):1865–71. 20197424 10.1093/eurheartj/ehq024

[14] Jenner R, Fatureto-Borges F, Costa-Hong V, Lopes HF, Teixeira SH, Marum E, et al. Association of obstructive sleep apnea with arterial stiffness and nondipping blood pressure in patients with hypertension. J Clin Hypertens (Greenwich). 2017;19(9):910–8. 28429850 10.1111/jch.13008 PMC8030757

[15] Saeed S, Romarheim A, Mancia G, Saxvig IW, Gulati S, Lehmann S, et al. Characteristics of hypertension and arterial stiffness in obstructive sleep apnea: A Scandinavian experience from a prospective study of 6408 normotensive and hypertensive patients. J Clin Hypertens (Greenwich). 2022;24(4):385–94. 35156757 10.1111/jch.14425 PMC8989758

[16] Lisan Q, van Sloten T, Boutouyrie P, Laurent S, Danchin N, Thomas F, et al. Sleep Apnea is Associated With Accelerated Vascular Aging: Results From 2 European Community-Based Cohort Studies. J Am Heart Assoc. 2021;10(15):e021318. 34308679 10.1161/JAHA.120.021318 PMC8475690

[17] Kwon Y, Logan J, Redline S, Duprez D, Jacobs DR Jr., Ouyang P, et al. Obstructive Sleep Apnea and Structural/Functional Properties of the Thoracic Ascending Aorta: The Multi-Ethnic Study of Atherosclerosis (MESA). Cardiology. 2019;142(3):180–8. 31189162 10.1159/000499500 PMC6712423

[18] Jagodzinski A, Johansen C, Koch-Gromus U, Aarabi G, Adam G, Anders S, et al. Rationale and Design of the Hamburg City Health Study. Eur J Epidemiol. 2020;35(2):169–81. 31705407 10.1007/s10654-019-00577-4 PMC7125064

[19] Bohnen S, Avanesov M, Jagodzinski A, Schnabel RB, Zeller T, Karakas M, et al. Cardiovascular magnetic resonance imaging in the prospective, population-based, Hamburg City Health cohort study: objectives and design. J Cardiovasc Magn resonance: official J Soc Cardiovasc Magn Reson. 2018;20(1):68. 10.1186/s12968-018-0490-7 PMC6151919 30244673

[20] Johns MW. A new method for measuring daytime sleepiness: the Epworth sleepiness scale. Sleep. 1991;14(6):540–5. 1798888 10.1093/sleep/14.6.540

[21] Loose S, Solou D, Strecker C, Hennemuth A, Hüllebrand M, Grundmann S, et al. Characterization of aortic aging using 3D multi-parametric MRI-long-term follow-up in a population study. Sci Rep. 2023;13(1):6285. 37072440 10.1038/s41598-023-33219-7 PMC10111081

[22] Ibrahim el SH, Johnson KR, Miller AB, Shaffer JM, White RD. Measuring aortic pulse wave velocity using high-field cardiovascular magnetic resonance: comparison of techniques. J Cardiovasc Magn resonance: official J Soc Cardiovasc Magn Reson. 2010;12(1):26. 10.1186/1532-429X-12-26 PMC2874535 20459799

[23] Wentland AL, Wieben O, François CJ, Boncyk C, Del Munoz A, Johnson KM, et al. Aortic pulse wave velocity measurements with undersampled 4D flow-sensitive MRI: comparison with 2D and algorithm determination. J Magn Reson imaging: JMRI. 2013;37(4):853–9. 23124585 10.1002/jmri.23877 PMC3566322

[24] Fielden SW, Fornwalt BK, Jerosch-Herold M, Eisner RL, Stillman AE, Oshinski JN. A new method for the determination of aortic pulse wave velocity using cross-correlation on 2D PCMR velocity data. J Magn Reson imaging: JMRI. 2008;27(6):1382–7. 18504758 10.1002/jmri.21387

[25] Taviani V, Patterson AJ, Graves MJ, Hardy CJ, Worters P, Sutcliffe MP, et al. Accuracy and repeatability of fourier velocity encoded M-mode and two-dimensional cine phase contrast for pulse wave velocity measurement in the descending aorta. J Magn Reson imaging: JMRI. 2010;31(5):1185–94. 20432355 10.1002/jmri.22143

[26] Harloff A, Zech T, Frydrychowicz A, Schumacher M, Schöllhorn J, Hennig J, et al. Carotid intima-media thickness and distensibility measured by MRI at 3 T versus high-resolution ultrasound. Eur Radiol. 2009;19(6):1470–9. 19214524 10.1007/s00330-009-1295-8

[27] McEvoy JW, McCarthy CP, Bruno RM, Brouwers S, Canavan MD, Ceconi C, et al. 2024 ESC Guidelines for the management of elevated blood pressure and hypertension: Developed by the task force on the management of elevated blood pressure and hypertension of the European Society of Cardiology (ESC) and endorsed by the European Society of Endocrinology (ESE) and the European Stroke Organisation (ESO). Eur Heart J. 2024;45(38):3912–4018. 39210715 10.1093/eurheartj/ehae178

[28] von Elm E, Altman DG, Egger M, Pocock SJ, Gøtzsche PC, Vandenbroucke JP. The Strengthening the Reporting of Observational Studies in Epidemiology (STROBE) statement: guidelines for reporting observational studies. Lancet. 2007;370(9596):1453–7. 18064739 10.1016/S0140-6736(07)61602-X

[29] Kim SW, Taranto-Montemurro L. When do gender differences begin in obstructive sleep apnea patients? J Thorac Dis. 2019;11(Suppl 9):S1147–9. 31245068 10.21037/jtd.2019.04.37 PMC6560552

[30] Eckert DJ, White DP, Jordan AS, Malhotra A, Wellman A. Defining phenotypic causes of obstructive sleep apnea. Identification of novel therapeutic targets. Am J Respir Crit Care Med. 2013;188(8):996–1004. 23721582 10.1164/rccm.201303-0448OC PMC3826282

[31] Coussa-Koniski ML, Saliba E, Welty FK, Deeb M. Epidemiological characteristics of obstructive sleep apnea in a hospital-based historical cohort in Lebanon. PLoS ONE. 2020;15(5):e0231528. 32413035 10.1371/journal.pone.0231528 PMC7228052

[32] Korcarz CE, Gepner AD, Peppard PE, Young TB, Stein JH. The effects of sleep-disordered breathing on arterial stiffness are modulated by age. Sleep. 2010;33(8):1081–5. 20815190 10.1093/sleep/33.8.1081 PMC2910537

[33] Williams B, Mancia G, Spiering W, Agabiti Rosei E, Azizi M, Burnier M, et al. 2018 ESC/ESH Guidelines for the management of arterial hypertension: The Task Force for the management of arterial hypertension of the European Society of Cardiology (ESC) and the European Society of Hypertension (ESH). Eur Heart J. 2018;39(33):3021–104. 30165516 10.1093/eurheartj/ehy339

[34] Punjabi NM. The epidemiology of adult obstructive sleep apnea. Proc Am Thorac Soc. 2008;5(2):136–43. 18250205 10.1513/pats.200709-155MG PMC2645248

[35] Franklin KA, Lindberg E. Obstructive sleep apnea is a common disorder in the population—a review on the epidemiology of sleep apnea. J Thorac Disease. 2015;7(8):1311–22. 26380759 10.3978/j.issn.2072-1439.2015.06.11 PMC4561280

[36] Iannella G, Pace A, Bellizzi MG, Magliulo G, Greco A, De Virgilio A et al. The Global Burden of Obstructive Sleep Apnea. Diagnostics (Basel). 2025;15(9). 10.3390/diagnostics15091088 PMC12071658 40361906

[37] Senaratna CV, Perret JL, Lodge CJ, Lowe AJ, Campbell BE, Matheson MC, et al. Prevalence of obstructive sleep apnea in the general population: A systematic review. Sleep Med Rev. 2017;34:70–81. 27568340 10.1016/j.smrv.2016.07.002

[38] Lisan Q, van Sloten T, Boutouyrie P, Laurent S, Danchin N, Thomas F, et al. Sleep Apnea is Associated With Accelerated Vascular Aging: Results From 2 European Community-Based Cohort Studies. J Am Heart Association. 2021;10(15):e021318. 10.1161/JAHA.120.021318 PMC8475690 34308679

[39] Pereira T, Correia C, Cardoso J. Novel Methods for Pulse Wave Velocity Measurement. J Med Biol Eng. 2015;35(5):555–65. 26500469 10.1007/s40846-015-0086-8 PMC4609308

[40] Grotenhuis HB, Westenberg JJ, Steendijk P, van der Geest RJ, Ottenkamp J, Bax JJ, et al. Validation and reproducibility of aortic pulse wave velocity as assessed with velocity-encoded MRI. J Magn Reson imaging: JMRI. 2009;30(3):521–6. 19711407 10.1002/jmri.21886

[41] McEvoy JW, McCarthy CP, Bruno RM, Brouwers S, Canavan MD, Ceconi C et al. 2024 ESC Guidelines for the management of elevated blood pressure and hypertension: Developed by the task force on the management of elevated blood pressure and hypertension of the European Society of Cardiology (ESC) and endorsed by the European Society of Endocrinology (ESE) and the European Stroke Organisation (ESO). Eur Heart J. 2024.

[42] Dart AM, Kingwell BA. Pulse pressure—a review of mechanisms and clinical relevance. J Am Coll Cardiol. 2001;37(4):975–84. 11263624 10.1016/s0735-1097(01)01108-1

[43] Maroules CD, Khera A, Ayers C, Goel A, Peshock RM, Abbara S, et al. Cardiovascular outcome associations among cardiovascular magnetic resonance measures of arterial stiffness: the Dallas heart study. J Cardiovasc Magn resonance: official J Soc Cardiovasc Magn Reson. 2014;16(1):33. 10.1186/1532-429X-16-33 PMC4031496 24886531

[44] Heinzer R, Vat S, Marques-Vidal P, Marti-Soler H, Andries D, Tobback N, et al. Prevalence of sleep-disordered breathing in the general population: the HypnoLaus study. Lancet Respir Med. 2015;3(4):310–8. 25682233 10.1016/S2213-2600(15)00043-0 PMC4404207

[45] Markl M, Wallis W, Brendecke S, Simon J, Frydrychowicz A, Harloff A. Estimation of global aortic pulse wave velocity by flow-sensitive 4D MRI. Magn Reson Med. 2010;63(6):1575–82. 20512861 10.1002/mrm.22353

[46] Rothman KJ. No adjustments are needed for multiple comparisons. Epidemiology. 1990;1(1):43–6. 2081237

[47] Deiseroth A, Streese L, Köchli S, Wüst RS, Infanger D, Schmidt-Trucksäss A, et al. Exercise and Arterial Stiffness in the Elderly: A Combined Cross-Sectional and Randomized Controlled Trial (EXAMIN AGE). Front Physiol. 2019;10:1119. 31551805 10.3389/fphys.2019.01119 PMC6738015

[48] Chalegre ST, Lins-Filho OL, Lustosa TC, França MV, Couto TLG, Drager LF, et al. Impact of CPAP on arterial stiffness in patients with obstructive sleep apnea: a meta-analysis of randomized trials. Sleep Breath. 2021;25(3):1195–202. 33094411 10.1007/s11325-020-02226-7

