# Supplementary material for: CMR-based aortic stiffness parameters in a symptomatic high risk obstructive sleep apnea cohort: results from the population-based Hamburg City Health Study
Source: BMC Cardiovasc Disord. 2026 Aug 13;26:691. doi: 10.1186/s12872-026-06450-z (PMC13471597; doi:10.1186/s12872-026-06450-z)
Supplement: Supplementary file 2 [file 12872_2026_6450_MOESM2_ESM.pdf]

Supplemental Material

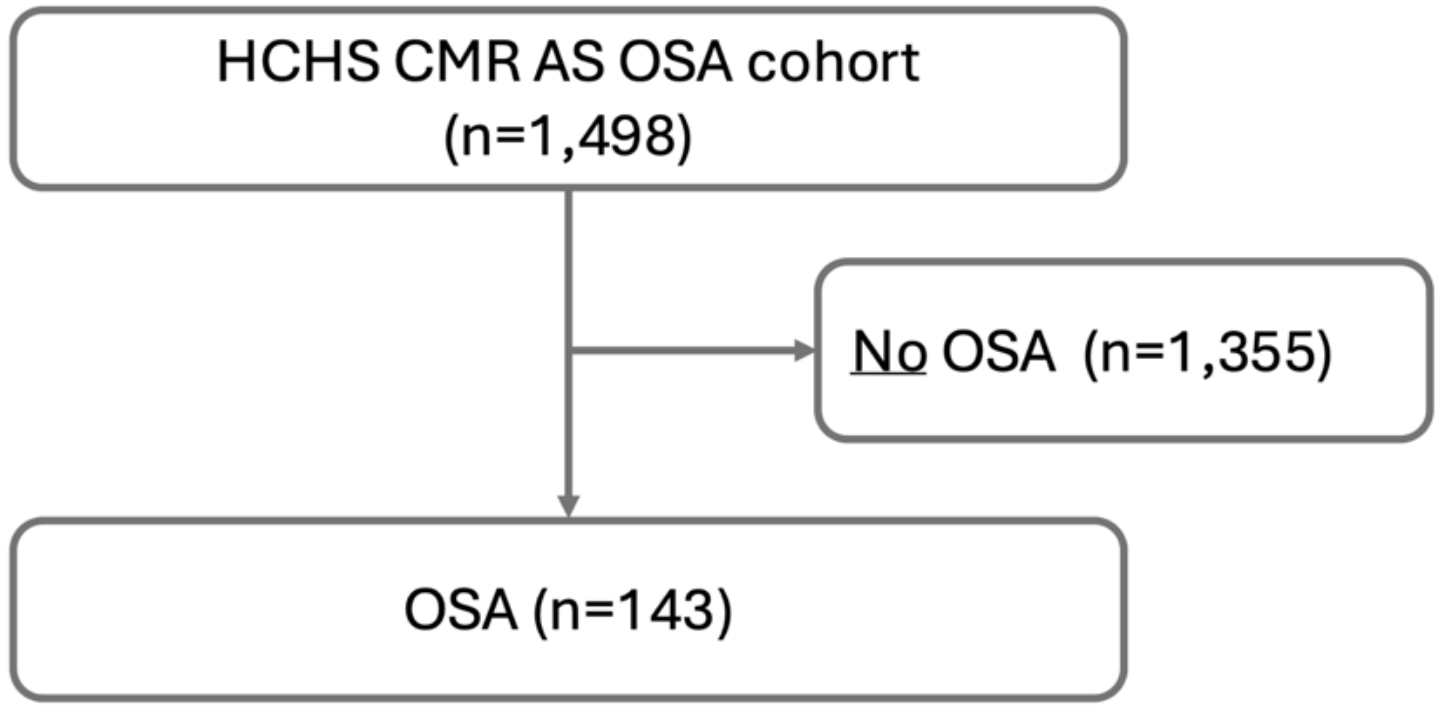

Supplemental Fig. 1 Flow Chart Study Population

1,498 participants underwent a cardiovascular magnetic resonance imaging scan, quantifying PWV and AD, PP assessment, as well as OSA data. 143 participants reported OSA and in 1,355 no OSA was reported. OSA was defined as self-reported OSA in the HCHS study interview or snoring with respiratory arrest and ESS >10.

HCHS = Hamburg City Health Study, CMR = cardiovascular magnetic resonance, AS = aortic stiffness, OSA = obstructive sleep apnea, ESS = Epworth Sleepiness Scale.

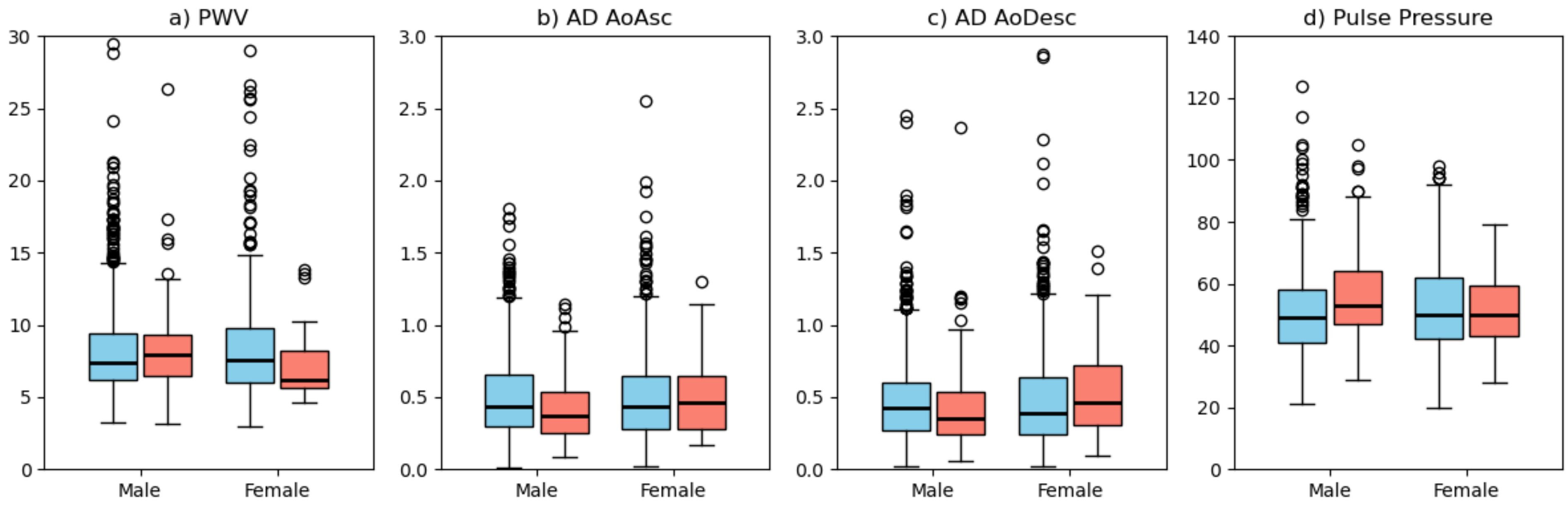

Supplemental Fig. 2 Boxplots PWV in [m/s] (p=0.412 and p=0.123) (a), AD AoAsc in [1/(10^3*kPa)] (p=0.006 and p=0.277) (b), AD AoDesc in [1/(10^3*kPa)] (p=0.015 and p=0.583) (c) and PP in [mmHg] (p<0.001 and p=0.706) (d) according to sex and OSA categories (No OSA (blue) vs. OSA (red))

PWV = pulse wave velocity, AD = aortic distensibility, AoAsc = ascending aorta, AoDesc = descending aorta, OSA = obstructive sleep apnea.

Supplemental Table 1 Study Population

	**HCHS CMR AS OSA cohort* (n=1,498)**
Age [years]	66.0 [58.0, 71.0]
Sex, females	584 (39.0)
Smoking (current and history)	983 (65.8)
BMI [kg/m^2]	26.3 [23.9, 29.3]
Systolic blood pressure before CMR [mmHg]	140.0 [128.0, 154.0]
Diastolic blood pressure before CMR [mmHg]	82.5 [76.5, 90.0]
Hypertension	625 (42.1)
Hyperlipidemia	388 (27.4)
Diabetes mellitus	136 (9.7)
Coronary artery disease	87 (5.9)
Myocardial infarction	51 (3.4)
Stroke	45 (3.0)
Heart failure	47 (3.9)
Creatinine [mg/dl]	0.87 [0.76, 0.99]
Medication	1173 (81.1)
Antihypertensive medication	606 (41.9)
Diuretics	30 (2.1)
ACE inhibitor and/or ARB	341 (23.6)
Betablocker	246 (17.0)
Anti-platelet aggregatory therapy	190 (13.1)
Aspirin	183 (12.7)
Antidepressants	84 (5.8)
Snoring	1041 (69.5)
Snoring Frequency	
Almost every day	351 (41.4)
3-5 times a week	180 (21.2)
1-2 times a week	199 (23.5)
1-2 times a month	71 (8.4)
Less than once a month	47 (5.5)

Respiratory Arrest	235 (15.7)
Respiratory Arrest Frequency	
Almost every day	78 (50.6)
3-5 times a week	16 (10.4)
1-2 times a week	21 (13.6)
1-2 times a month	11 (7.1)
Less than once a month	28 (18.2)
ESS>10	197 (13.2)
Aortic Stiffness Parameter	
PWV [m/s]	7.45 [6.10, 9.60]
AD AoAsc [1/(10^3*kPa)]	0.40 [0.26, 0.61]
AD AoDesc [1/(10^3*kPa)]	0.43 [0.28, 0.65]
PP [mmHg]	50.0 [42.0, 60.0]

Continuous data are median and interquartile range. Categorical data are absolute numbers with percentages. CMR = cardiovascular magnetic resonance, AS = aortic stiffness, OSA = obstructive sleep apnea, BMI = body mass index, ACE = angiotensin converting enzyme, ARB = angiotensin II receptor blocker, PWV = pulse wave velocity, AD = aortic distensibility, AoAsc = ascending aorta, AoDesc = descending aorta, PP = pulse pressure, ESS = Epworth Sleepiness Scale.

* Including all participants with CMR-based PWV and AD assessment, PP assessment and OSA data.

Supplemental Table 2 Study population according to OSA status and age categories

	**CMR AS cohort <u>without</u> OSA 45-54 years (n=239)**	**CMR AS cohort <u>with</u> OSA 45-54 years (n=19)**	***p-value***	**CMR AS cohort <u>without</u> OSA 55-64 years (n=369)**	**CMR AS cohort <u>with</u> OSA 55-64 years (n=41)**	***p-value***	**CMR AS cohort <u>without</u> OSA ≥65 years (n=747)**	**CMR AS cohort <u>with</u> OSA ≥65 years (n=83)**	***p-value***
Sex, females	107 (44.8)	6 (31.6)	0.381	161 (43.6)	10 (24.4)	0.028	286 (38.3)	14 (16.9)	< 0.001
Smoking (current and history)	147 (61.8)	10 (52.6)	0.588	237 (64.4)	31 (75.6)	0.208	500 (67.0)	58 (70.7)	0.497
BMI [kg/m^2]	25.6 [23.1, 28.9]	29.3 [25.2, 33.9]	0.003	26.5 [23.8, 29.0]	29.9 [25.5, 34.7]	< 0.001	26.2 [23.9, 28.9]	28.8 [26.6, 32.5]	< 0.001
Systolic blood pressure before CMR [mmHg]	132.0 [122.0, 143.0]	137.0 [126.0, 146.0]	0.469	136.0 [126.0, 152.0]	138.0 [128.0, 150.0]	0.456	144.0 [133.0, 158.0]	143.0 [134.0, 154.0]	0.891
Diastolic blood pressure before CMR [mmHg]	83.0 [77.0, 90.5]	84.5 [80.8, 89.2]	0.331	84.0 [79.0, 91.5]	84.5 [78.5, 90.2]	0.960	81.5 [75.5, 89.0]	80.0 [74.5, 86.5]	0.340
Hypertension	44 (18.7)	8 (42.1)	0.033	147 (40.1)	25 (61.0)	0.010	345 (46.7)	56 (67.5)	< 0.001
Hyperlipidemia	25 (11.6)	4 (21.1)	0.405	88 (26.0)	11 (28.9)	0.699	231 (31.8)	29 (36.2)	0.416
Diabetes Mellitus	10 (4.7)	2 (10.5)	0.584	24 (7.0)	4 (10.5)	0.651	86 (11.9)	10 (13.2)	0.902
Coronary artery disease	4 (1.7)	3 (15.8)	0.004	15 (4.1)	2 (4.9)	1.000	54 (7.4)	9 (10.8)	0.366
Myocardial infarction	4 (1.7)	1 (5.3)	0.822	9 (2.5)	2 (4.9)	0.686	30 (4.0)	5 (6.0)	0.572
Stroke	2 (0.8)	0 (0.0)	1.000	8 (2.2)	1 (2.4)	1.000	33 (4.4)	1 (1.2)	0.272
Heart failure	6 (3.0)	0 (0.0)	1.000	8 (2.7)	1 (2.7)	1.000	26 (4.5)	6 (9.2)	0.171
Creatinine [mg/dl]	0.85 [0.75, 0.96]	0.90 [0.81, 1.10]	0.052	0.85 [0.75, 0.96]	0.91 [0.82, 1.00]	0.061	0.89 [0.77, 1.00]	0.93 [0.80, 1.10]	0.082
Medication	163 (74.1)	14 (73.7)	1.000	275 (78.1)	36 (90.0)	0.121	614 (83.7)	71 (87.7)	0.439
Antihypertensive medication	40 (18.2)	6 (31.6)	0.264	133 (37.8)	22 (55.0)	0.035	351 (47.8)	54 (66.7)	0.001
Diuretics	0 (0.0)	1 (5.3)	0.119	5 (1.4)	1 (2.5)	1.000	21 (2.9)	2 (2.5)	1.000
ACE inhibitor and/or ARB	28 (12.7)	4 (21.1)	0.502	75 (21.3)	15 (37.5)	0.021	186 (25.3)	33 (40.7)	0.003
Betablocker	11 (5.0)	0 (0.0)	0.669	49 (13.9)	8 (20.0)	0.425	159 (21.7)	19 (23.5)	0.711
Anti-platelet aggregatory therapy	7 (3.2)	2 (10.5)	0.324	21 (6.0)	6 (15.0)	0.071	132 (18.0)	22 (27.2)	0.045
Aspirin	7 (3.2)	2 (10.5)	0.324	21 (6.0)	6 (15.0)	0.071	125 (17.0)	22 (27.2)	0.024
Antidepressants	8 (3.6)	1 (5.3)	1.000	30 (8.5)	4 (10.0)	0.986	34 (4.6)	7 (8.6)	0.194

Snoring	154 (64.4)	19 (100.0)	0.003	255 (69.1)	39 (95.1)	< 0.001	495 (66.3)	79 (95.2)	< 0.001
Snoring Frequency									
Almost every day	49 (37.4)	16 (94.1)	< 0.001	81 (39.3)	34 (91.9)	< 0.001	120 (30.9)	51 (73.9)	< 0.001
3-5 times a week	25 (19.1)	1 (5.9)	0.314	43 (20.9)	1 (2.7)	0.016	101 (26.0)	9 (13.0)	0.030
1-2 times a week	31 (23.7)	0 (0.0)	0.053	44 (21.4)	2 (5.4)	0.040	116 (29.9)	6 (8.7)	< 0.001
1-2 times a month	16 (12.2)	0 (0.0)	0.267	25 (12.1)	0 (0.0)	0.052	27 (7.0)	3 (4.4)	0.587
Less than once a month	10 (7.6)	0 (0.0)	0.505	13 (6.3)	0 (0.0)	0.240	24 (6.2)	0 (0.0)	0.067
Respiratory Arrest	18 (7.5)	18 (94.7)	< 0.001	29 (7.9)	39 (95.1)	< 0.001	56 (7.5)	75 (90.4)	< 0.001
Respiratory Arrest Frequency									
Almost every day	1 (14.3)	6 (75.0)	0.067	5 (20.8)	17 (81.0)	< 0.001	5 (12.8)	44 (80.0)	< 0.001
3-5 times a week	2 (28.6)	1 (12.5)	0.897	3 (12.5)	1 (4.8)	0.700	6 (15.4)	3 (5.5)	0.209
1-2 times a week	2 (28.6)	0 (0.0)	0.388	2 (8.3)	0 (0.0)	0.530	11 (28.2)	6 (10.9)	0.061
1-2 times a month	0 (0.0)	1 (12.5)	1.000	4 (16.7)	0 (0.0)	0.151	6 (15.4)	0 (0.0)	0.010
Less than once a month	2 (28.6)	0 (0.0)	0.388	10 (41.7)	3 (14.3)	0.091	11 (28.2)	2 (3.6)	0.002
ESS>10	34 (14.2)	12 (63.2)	< 0.001	50 (13.6)	20 (48.8)	< 0.001	59 (7.9)	22 (26.5)	< 0.001
Aortic stiffness parameter									
PWV [m/s]	5.43 [4.69, 6.28]	5.63 [4.93, 6.59]	0.321	6.84 [5.82, 8.17]	6.73 [6.05, 8.31]	0.560	8.61 [7.18, 10.80]	8.29 [6.90, 10.00]	0.063
AD AoAsc [1/(10^3*kPa)]	0.64 [0.46, 0.87]	0.57 [0.45, 0.69]	0.763	0.47 [0.33, 0.71]	0.47 [0.28, 0.65]	0.463	0.34 [0.23, 0.48]	0.31 [0.20, 0.44]	0.234
AD AoDesc [1/(10^3*kPa)]	0.70 [0.51, 0.92]	0.61 [0.46, 0.91]	0.461	0.47 [0.33, 0.67]	0.47 [0.29, 0.75]	0.947	0.36 [0.25, 0.53]	0.34 [0.23, 0.46]	0.132
PP [mmHg]	43.0 [37.0, 50.0]	48.5 [41.2, 52.0]	0.091	48.0 [40.0, 56.0]	50.0 [40.8, 60.5]	0.172	53.0 [45.0, 63.0]	59.0 [48.0, 64.0]	0.041

Continuous data are median and interquartile range. Categorical data are absolute numbers with percentages. CMR = cardiovascular magnetic resonance, AS = aortic stiffness, OSA = obstructive sleep apnea, BMI = body mass index, ACE = angiotensin converting enzyme, ARB = angiotensin II receptor blocker, PWV = pulse wave velocity, AD = aortic distensibility, AoAsc = ascending aorta, AoDesc = descending aorta, PP = pulse pressure, ESS = Epworth Sleepiness Scale.

Supplemental Table 3 Regression analysis of OSA with PWV, AD and PP without adjustment

	PWV model		AD AoAsc model		AD AoDesc model		PP model	
	**OR [95% CI]**	***p-value***	**OR [95% CI]**	***p-value***	**OR [95% CI]**	***p-value***	**OR [95% CI]**	***p-value***
**PWV/AD/PP variable**	0.986 [0.934, 1.040]	0.597	0.734 [0.412, 1.307]	0.293	0.492 [0.257, 0.943]	0.033	1.019 [1.007, 1.031]	0.002

OSA = obstructive sleep apnea, PWV = pulse wave velocity, AD = aortic distensibility, AoAsc = ascending aorta, AoDesc = descending aorta, PP = pulse pressure, OR = odds ratio.

Supplemental Table 4 Regression analysis of OSA with PWV, AD and PP after adjustment for age and sex

	PWV model		AD AoAsc model		AD AoDesc model		PP model	
	**OR [95% CI]**	***p-value***	**OR [95% CI]**	***p-value***	**OR [95% CI]**	***p-value***	**OR [95% CI]**	***p-value***
**PWV/AD/PP variable**	0.975 [0.916, 1.039]	0.436	0.757 [0.396, 1.448]	0.401	0.474 [0.231, 0.973]	0.042	1.020 [1.007, 1.033]	0.002
**Age**	1.010 [0.986, 1.034]	0.407	1.002 [0.979, 1.025]	0.895	0.996 [0.974, 1.019]	0.757	0.998 [0.975, 1.022]	0.877
**Sex**	0.388 [0.256, 0.589]	< 0.001	0.385 [0.254, 0.585]	< 0.001	0.385 [0.253, 0.584]	< 0.001	0.361 [0.233, 0.56]	< 0.001

OSA = obstructive sleep apnea, PWV = pulse wave velocity, AD = aortic distensibility, AoAsc = ascending aorta, AoDesc = descending aorta, OR = odds ratio, PP = pulse pressure.

Supplemental Table 5 Regression analysis of OSA with PWV, AD and PP after adjustment for age, sex, obesity (BMI >30 kg/m^2) and hypertension

	PWV model		AD AoAsc model		AD AoDesc model		PP model	
	**OR [95% CI]**	***p-value***	**OR [95% CI]**	***p-value***	**OR [95% CI]**	***p-value***	**OR [95% CI]**	***p-value***
**PWV/AD/PP variable**	0.975 [0.914, 1.039]	0.427	0.763 [0.392, 1.487]	0.427	0.567 [0.274, 1.172]	0.126	1.015 [1.002, 1.028]	0.026
**Age**	1.005 [0.980, 1.030]	0.707	0.996 [0.972, 1.021]	0.768	0.993 [0.970, 1.018]	0.597	0.999 [0.975, 1.023]	0.909
**Sex**	0.369 [0.241, 0.565]	< 0.001	0.369 [0.241, 0.565]	< 0.001	0.369 [0.241, 0.565]	< 0.001	0.354 [0.227, 0.553]	< 0.001
**Obesity (BMI >30 kg/m^2)**	3.109 [2.129, 4.539]	< 0.001	3.100 [2.123, 4.525]	< 0.001	3.031 [2.074, 4.428]	< 0.001	2.878 [1.94, 4.271]	< 0.001
**Hypertension**	2.014 [1.381, 2.937]	< 0.001	2.019 [1.384, 2.944]	< 0.001	2.019 [1.385, 2.944]	< 0.001	1.745 [1.179, 2.583]	0.005

OSA = obstructive sleep apnea, PWV = pulse wave velocity, AD = aortic distensibility, AoAsc = ascending aorta, AoDesc = descending aorta, OR = odds ratio, PP = pulse pressure, BMI = Body mass index.

Supplemental Table 6 Variance Inflation Factors (VIF) for regression analysis of OSA with PWV, AD and PP after adjustment for age, sex, cardiovascular risk factors and diseases and antihypertensive medication.

	PWV model	AD AoAsc model	AD AoDesc model	PP model
**PWV/AD/PP variable**	1.283	1.206	1.174	1.159
**Age**	1.356	1.279	1.243	1.187
**Sex**	1.018	1.016	1.017	1.02
**Obesity (BMI >30 kg/m^2)**	1.049	1.049	1.055	1.056
**Hypertension**	2.869	2.859	2.867	2.922
**Hyperlipidemia**	1.184	1.184	1.184	1.189
**Smoking**	1.003	1.004	1.003	1.009
**Diabetes mellitus**	1.08	1.076	1.076	1.078
**CAD**	1.426	1.421	1.423	1.407
**Myocardial infarction**	1.349	1.348	1.348	1.333
**Antihypertensive medication**	2.989	2.983	2.997	3.009

OSA = obstructive sleep apnea, PWV = pulse wave velocity, AD = aortic distensibility, AoAsc = ascending aorta, AoDesc = descending aorta, OR = odds ratio, PP = pulse pressure, BMI = body mass index, CAD = coronary artery disease.

Supplemental Table 7 Likelihood ratio tests of the assumed linear terms to restricted cubic splines of degree 4 (p-values).

	PWV model	AD AoAsc model	AD AoDesc model	PP model
**PWV/AD/PP variable**	0.575	0.135	0.862	0.517
**Age**	0.945	0.946	0.951	0.980

OSA = obstructive sleep apnea, PWV = pulse wave velocity, AD = aortic distensibility, AoAsc = ascending aorta, AoDesc = descending aorta, PP = pulse pressure.
